# Respiratory viruses associated with severe acute respiratory infection in children aged <5 years at a tertiary care hospital in Delhi, India during 2013–15

**DOI:** 10.7189/jogh.14.04230

**Published:** 2024-11-08

**Authors:** Satinder Aneja, Varinder Singh, Venkatesh Vinayak Narayan, Mayuri Gohain, Avinash Choudekar, Bharti Gaur, Katherine Roguski DeBord, Brett Whitaker, Anand Krishnan, Shobha Broor, Siddhartha Saha, A Danielle Iuliano

**Affiliations:** 1 Kalawati Saran Children’s Hospital & Lady Hardinge Medical College, New Delhi, India; 2 Influenza Program, US Centers for Disease Control and Prevention – Delhi office, New Delhi, India; 3 All India Institute of Medical Sciences, New Delhi, India; 4 US Centers for Disease Control and Prevention, Atlanta, Georgia, USA

## Abstract

**Background:**

With the increased availability of licensed vaccines for respiratory viruses such as severe acute respiratory syndrome coronavirus 2, respiratory syncytial virus (RSV), and influenza virus, a better understanding of the viral aetiology of severe acute respiratory infections (SARI) among children could help in optimising the use of these vaccines. We conducted a study among children aged <5 years hospitalised with SARI at a tertiary care children’s hospital in north India and tested for common respiratory pathogens.

**Methods:**

We randomly enrolled eligible SARI cases aged <5 years from August 2013 to July 2015. SARI cases were defined as either <7-day history of fever with cough or in children aged eight days to three months, a physician diagnosis of acute lower respiratory infection requiring hospitalisation. We also enrolled an age-group matched control without any acute illness in a 2:1 ratio from the outpatient clinic within 24 hours of case enrolment. Nasopharyngeal and/or oropharyngeal swabs were collected and tested using TaqMan Array Cards, a real-time reverse transcription polymerase chain reaction-based multi-pathogen testing platform for selected respiratory viruses among the enrolled cases and controls. We compared the prevalence of each pathogen among cases and controls using the χ^2^ (χ^2^) or Fisher exact test (*P* < 0.05). We used logistic regression to estimate adjusted odds ratios (aORs) which were then used to calculate aetiologic fractions (EFs).

**Results:**

We enrolled 840 cases and 419 outpatient controls. Almost half of the individuals in the whole sample were aged <6 months (n = 521, 41.4%). Females made up 33.7% of cases and 37.2% of controls. Viral detections were more common among cases (69%, 95% confidence interval (CI) = 66, 73) compared to controls (33%; 95% CI = 29, 38) (*P* < 0.01). RSV (n = 257, 31%; 95% CI = 28, 34%) was the most common virus detected among cases. Influenza A was detected among 24 (3%; 95% CI = 2, 4%), and influenza B among 5 (1%; 95% CI = 0, 1%) cases. The association between the virus and SARI was strongest for RSV (aOR = 23; 95% CI = 12, 47; EF = 96%). Antivirals were administered to 1% of SARI cases while 78% received antibiotics.

**Conclusions:**

Using a multi-pathogen molecular detection method, we detected respiratory viruses among more than two-thirds of children aged <5 years admitted with SARI in the Delhi tertiary care children’s hospital. The guidelines for preventing and managing SARI cases among children could be optimised further with the improved availability of antivirals and vaccines.

Globally, acute lower respiratory infections (ALRI) are the most common infectious cause of death among children <5 years, with 99% of these deaths occurring in low- and middle-income countries (LMICs) [1–3]. The overall progress in reducing ALRI in LMICs has been supported through increased availability and usage of pneumococcal conjugate and *Haemophilus influenzae* type b vaccines. This, along with improved molecular diagnostic testing for respiratory viruses, has greatly aided in the identification and increased recognition of viral causes for ALRI [4–6].

Worldwide, more than half of ALRIs are attributed to respiratory viruses [6]. Studies across countries among children hospitalised with respiratory illness have found the respiratory syncytial virus (RSV) to be the most common viral pathogen, with other frequently detected viruses being the human metapneumovirus, parainfluenza viruses, influenza viruses, and adenoviruses [4,7,8]. Estimates of the global respiratory virus burden also show a disproportionately higher contribution from LMICs, pointing to the low usage of vaccines and antivirals in these countries [2,9,10].

The coronavirus disease 2019 (COVID-19) pandemic has refocussed attention towards the control of respiratory viruses, providing an incentive to the production of related vaccines, monoclonal antibodies, and therapeutics [11–13]. To better understand the value and utility of these products in the pipeline, data on the aetiology of respiratory viruses among hospitalised children could prove helpful. For India, few studies have documented a high proportion of RSV among children with ALRI, but with the emergence of monoclonal antibodies and the maternal vaccine against RSV, more RSV burden data are needed for informing policy [14,15]. Earlier studies in India have estimated influenza- and RSV-associated hospitalisation and death rates among children <5 years [16–18], but other respiratory pathogens have not been evaluated. As ALRI frequently presents with similar clinical features, additional studies could inform clinical practice by guiding the aetiology, especially among those hospitalised with severe acute respiratory infections (SARI). Appropriate testing for viruses, in turn, could support the use of appropriate antivirals which, in conjunction with vaccines, especially for RSV and influenza, could prevent overuse of antibiotics for ALRI management [11,19–21]. Few studies from hospitals in India have reported the prevalence of respiratory viruses of SARI among children <5 years [22,23] although the lack of controls in these studies makes the aetiology attribution difficult. Multi-site studies like the Pneumonia Etiology Research for Child Health (PERCH) or Global Approach to Biological Research, Infectious diseases and Epidemics in Low-income countries (GABRIEL), meanwhile, enrolled controls to facilitate aetiological attribution and to account for the presence of commensals/carriage [6,25]. Therefore, we assessed the prevalence of 15 respiratory viruses using upper respiratory tract samples from children <5 years hospitalised with SARI and those without acute illness. We estimated the aetiologic fraction (EF) for selected respiratory viruses to better understand the causative potential for individual pathogens.

## METHODS

### Study design and setting

We prospectively enrolled children aged <5 years with SARI at a tertiary care children’s hospital from August 2013 to July 2015. We used the World Health Organization (WHO) SARI case definition for children aged three months to five years as acute respiratory illness requiring hospitalisation with a history of fever or measured fever (≥38°C) and cough with onset within the last seven days from August 2013 to July 2014 [26], as well as the revised case-definition with onset within the last 10 days from August 2014 to July 2015 [27]. We selected these definitions specifically as they had been available during the study years. For children aged eight days to three months, we considered children with a physician-diagnosed acute lower respiratory infection and requiring hospitalisation as SARI irrespective of the signs and symptoms.

We conducted this study in New Delhi, northern India, at the Kalawati Saran Children’s Hospital associated with the Government Medical College, which is a tertiary care public hospital and one of the the largest government hospitals for children that covers Delhi and adjacent areas in north India and hosts extensive outpatient and inpatient facilities. This project was undertaken in collaboration with All India Institute of Medical Sciences Delhi, which provided the laboratory and data management support.

### Sample size

To estimate the sample size, we reviewed the expected frequency of rhinovirus, a viral pathogen most likely to cause colonisation or subclinical infection. Available data suggests that the prevalence of subclinical rhinovirus infection among children <5 years old in Thailand was 17.6%, while 29% of children hospitalised for ALRI tested positive for rhinovirus [28]. Based on this, to detect an 11% difference in the prevalence of rhinovirus among cases compared to controls, a site would need to enrol at least 400 cases and 200 controls (power of 85% and alpha of 0.05) over a year.

### Enrolment of participants

Trained study physicians visited the hospital’s paediatric wards daily and, using a pre-designed checklist, screened children aged <5 years who were admitted with respiratory complaints and hospitalised in the previous 24 hours for SARI. Among children who met the SARI case definition, we enrolled the first two eligible SARI cases who were admitted in the previous 24 hours to ensure uniformity in the enrolment process. For every two enrolled cases, we enrolled an age-group-matched control without any acute illness from the outpatient clinic within a day of case enrolment. Eligible controls were children with non-infectious diseases like malnutrition, other chronic diseases, or those who had come for immunisation; they must not have had any respiratory symptoms in the past 10 days or not have been hospitalised for any cause in the past 14 days. This 2:1 ratio of case and control enrolment was chosen to optimise available resources. Age groups used for the matching of controls were eight days to six months, 6–12 months, 12–23 months, and 24–59 months. To avoid detection of hospital-acquired infection, children with an episode of SARI in the prior 30 days or admission to a different hospital for more than 48 hours before referral or hospitalisation for any cause in the previous 14 days were excluded from being either a case or control.

The adoption of a well-designed research protocol and tools helped standardise training and data collection through study physicians. Regular supervision and review by study collaborators helped minimise and overcome any involuntary biases during the study. The child’s parent or guardian gave written informed consent before enrolment.

### Data and specimen collection

The study physician interviewed the parent or guardian using a structured questionnaire to capture demographic details and household characteristics. We collected clinical data including illness details, comorbidities, and treatment history through hospital records. We followed the enrolled SARI cases until discharge or death to record the outcome of the hospitalisation. Nasopharyngeal and/or oropharyngeal swabs were collected at enrolment and stored at −72°C until testing. For children aged <6 months, only a nasopharyngeal swab was collected. The specimens were transported in a viral transport medium (HiMedia Laboratories, Mumbai, India) with ice, to the laboratory on the same day.

### Laboratory testing

We conducted laboratory testing at the virology laboratory of the All India Institute of Medical Sciences, New Delhi, India using TaqMan Array Card, a real-time reverse transcription polymerase chain reaction-based multi-pathogen testing platform. Specimens were stored at −72°C if not tested within 48 hours of collection.

Extraction was carried out with Magna Pure Compact (Roche Diagnostics, Indianapolis, USA). Each TaqMan Array Card tested six specimens using a panel of 31 individual real-time reverse transcription polymerase chain reaction assays, targeting 16 viruses and 14 bacteria [29]. The used primers and probes were designed and synthesised by the Centers for Disease Control and Prevention (CDC) through the International Resource Reagent (information available from CDC upon request) [30]. Since we used upper respiratory tract specimens only, we limited our analyses to respiratory viruses. We assessed the prevalence of 15 respiratory viruses: influenza virus types A and B; RSV; metapneumovirus (hMPV); parainfluenza viruses (PIVs) types 1–4, human rhinoviruses; adenoviruses; enteroviruses; and human coronaviruses types 229E, NL63, OC43 and HKU1.

The reaction mixes for each specimen were prepared in a clean assay setup room, using AgPath-ID One-step reverse transcription polymerase chain reaction assays kits as per the CDC protocol [31]. All TaqMan Array Card runs were performed on the Thermo Fisher Scientific Inc., ViiA 7 real-time polymerase chain reaction instrument using the AgPath-ID One-Step reverse transcription polymerase chain reaction kit (Thermo Fisher Scientific Inc., Foster City, California, USA). A cycle threshold value of 43 was considered a cut-off value for interpretation as positive or negative [29].

### Data analysis

We entered all data in Epi-Info (CDC, Atlanta, Georgia, USA) and analysed it using STATA, version 16/SE (StataCorp LLC, College Station, Texas, USA). We compared the prevalence of each virus detection among cases and controls using χ^2^ (χ^2^) or Fisher exact test (*P* < 0.05). We used multivariable logistic regression models for select viruses that were significantly more common in cases (or controls) to estimate the adjusted odds ratio (aOR) of detecting the virus. Based on a literature review, for our aOR calculation, we considered the presence of a virus as the exposure and the odds of a positive detection of a specific virus between SARI cases and asymptomatic controls, comparing them after adjusting for potential confounders including age, gender, month of collection, and viral co-detection [32]. We used aORs to calculate the virus-specific EF, which is the proportion of cases in the exposed population in which the exposure has played a possible causal role in disease development by using the following equation: EF = (aOR - 1) / aOR.

## RESULTS

### Demographic and household risk factors

During the study period (August 2013 to July 2015), we observed 17 880 admissions of children <5 years old at the study hospital. Among these, 6737 (38%) were for all-cause respiratory illnesses. Of all-cause respiratory hospitalisations, 4015 (60%) children met the SARI case definition and were eligible for enrolment. We enrolled 840 (21%) children with SARI and 419 age-group-matched outpatient controls without acute illness. Nearly two-thirds of participants (66% of cases and 65% of controls) were aged <12 months (**Table 1**). Most enrolled children (66% of cases and 63% of controls) were males.

**Table 1:** Demographic characteristics of SARI cases and asymptomatic outpatient controls among children aged <5 y in Delhi (2013–15)*

	All SARI (n = 840)	Controls (n = 419)
Age group in months		
*0–2*	188 (22.4)	117 (27.9)
*3–5*	157 (18.7)	59 (14.1)
*6–11*	207 (24.6)	98 (23.4)
*12–23*	179 (21.3)	90 (21.5)
*≥24*	109 (13.0)	55 (13.1)
Sex (biological)		
*Males*	557 (66.3)	263 (62.8)
*Females*	283 (33.7)	156 (37.2)
Household characteristics		
*Household members, MD (IQR)*	5 (4–8)	6 (4–8)
*Children in house, MD (IQR)*	2 (2–3)	2 (1–3)
*Households >2.5 persons/room*†	679 (80.1)	292 (69.7)
*Tobacco smoker(s) in household*†	241 (28.7)	86 (20.5)
History of recent ARI in family†	222 (26.4)	10 (2.4)
Cooking fuel used‡		
*Liquefied petroleum gas*	768 (91.4)	396 (94.5)
*Electricity*	1 (0.1)	0 (0.0)
*Kerosene*	8 (1.0)	2 (0.5)
*Biomass fuels (firewood/charcoal/crop waste/dung)*†	122 (14.5)	42 (10.0)

ARI – acute respiratory illness, MD – median, IQR – interquartile range, SARI – severe acute respiratory illness

*Presented as n (%) unless specified otherwise.

†Significantly higher proportion among cases than controls (*P* < 0.05 per χ^2^).

‡Multiple options possible.

Although the average household size of cases (median (MD) = 5, interquartile range (IQR) = 4–8) and controls (MD = 6, IQR = 4–8) were comparable, cases (80%) were more likely to live in overcrowded homes (i.e. >2.5 persons per room) than controls (70%) (*P* < 0.01) (**Table 1**). More cases (29%) than controls (21%) reported the presence of current smokers in the household (*P* < 0.01). Although >90% of households overall used liquefied petroleum gas for cooking fuel, cooking with biomass fuels was more common among case households (15%) compared to control households (10%) (*P* < 0.01). Significantly, more than one-fourth of case households (26%) reported the occurrence of acute respiratory illness among a family member compared to 2% among control households (*P* < 0.01).

### Clinical characteristics, comorbidities, and diagnosis

Diagnosed comorbidities were not common among cases (**Table 2**). Heart disease (n = 21, 3%) and neurological conditions (n = 21, 3%) were the most frequently reported conditions. The median time between onset of illness and hospital admission was three days (IQR = 2–4), while median interval between onset and specimen collection was four days (IQR = 2–5) from August 2013 to July 2014 with an older seven-day SARI case definition, and four days (IQR = 3–6) from August 2014 to July 2015 with revised 10-day SARI case definition. Besides cough (>99%) and fever (>85%), which were a part of the definition, the most common clinical symptom was shortness of breath (>94%) across virus categories (**Table 2**; Table S1 in the **Online Supplementary Document**) The common clinical diagnoses for SARI cases were acute bronchiolitis (n = 325), pneumonia (n = 250), and lower respiratory infection with wheezing (n = 157).

**Table 2:** Demographic and clinical characteristics of children <5 y hospitalised with SARI at a tertiary care centre in Delhi, India (2013–15)*

	All SARI (n = 840)	Any virus (n = 583)		RSV positive (n = 257)		Influenza positive (n = 28)
Demographic characteristics						
*Infants (<1 y)*	552 (65.7)		391 (67.1)		210 (81.7)	15 (53.6)
*Males*	557 (66.3)		395 (67.8)		176 (68.5)	15 (53.6)
*Breastfed (for infants <1 y)*	338 (61.3)		247 (63.3)		140 (66.7)	6 (40.0)
Clinical and treatment history						
*ARI in a family member*	222 (26.4)		159 (27.3)		67 (26.1)	11 (39.3)
*Any heart or neurological disease*	42 (5.0)		23 (4.0)		12 (4.7)	2 (7.1)
*Received previous care before hospitalisation*	702 (83.6)		492 (84.4)		227 (88.3)	28 (100)
*Delay in admission (>4 d)*	202 (24.1)		125 (21.4)		37 (14.4)	15 (53.6)
Symptoms and signs						
*Cough*	836 (99.5)		582 (99.8)		257 (100)	28 (100)
*Fever*	767 (91.3)		534 (91.6)		218 (84.8)	28 (100)
*Breathlessness*	815 (97.0)		568 (97.4)		256 (99.6)	28 (100)
*Noisy breathing*	688 (82.3)		479 (82.7)		221 (86.0)	17 (63.0)
*Nasal discharge/congestion*	461 (54.9)		332 (57.0)		130 (50.6)	21 (75.0)
*Diarrhoea*	152 (18.1)		104 (17.8)		49 (19.1)	3 (10.7)
*Earache*	13 (1.6)		9 (1.5)		3 (1.2)	0 (0.0)
*Rash*	29 (3.5)		9 (1.5)		2 (0.8)	0 (0.0)
*Tachypnoea*	606 (72.1)		419 (71.9)		202 (78.6)	15 (53.6)
*Wheeze*	22 (2.6)		15 (2.6)		6 (2.3)	1 (3.6)
*Any sign of respiratory distress*†	652 (77.6)		457 (78.4)		223 (86.8)	20 (71.4)
*Lethargy*	628 (74.8)		447 (76.7)		216 (84.1)	21 (75.0)
Laboratory detection of >1 virus	101 (12.0)		101 (17.3)		40 (15.6)	11 (39.3)
Clinical management						
*Antibiotics*	651 (78.0)		464 (79.6)		210 (81.7)	24 (85.7)
*Antivirals*	7 (1.0)		4 (0.7)		2 (0.8)	0 (0.0)
*Supplemental O_2_*	516 (62.0)		363 (62.4)		163 (63.4)	19 (67.9)
*Mechanical ventilation*	19 (3.0)		13 (2.3)		5 (2.0)	2 (7.1)
*Outcome (n = 700)*	9 (1.0)		7 (1.4)		4 (1.9)	0 (0.0)

SARI – severe acute respiratory illness

*All values are presented as n (%).

†Defined as the presence of any of the signs of chest in-drawing, nasal flaring, cyanosis, stridor, head bobbing, or accessory muscle use.

### Respiratory virus detection and seasonal trends

**Figure 1:**
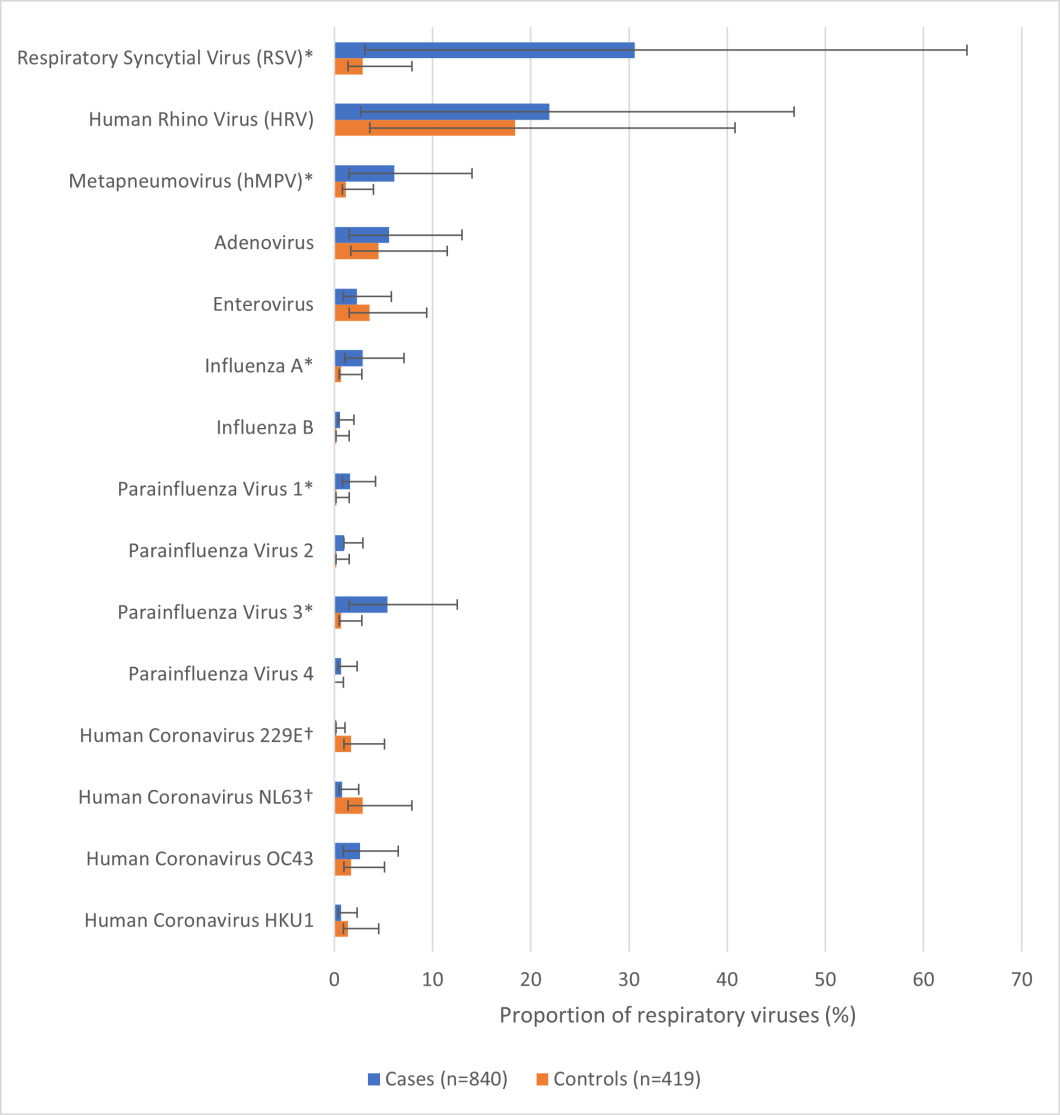
Prevalence of respiratory viruses among SARI cases and asymptomatic controls aged <5 years in Delhi (2013–15). *Pathogens significantly more common in cases than controls (*P* < 0.05). †Pathogens significantly more common in controls than cases (*P* < 0.05)

We detected one or more respiratory viruses among 69% of cases (95% confidence interval (CI) = 66, 73) compared to 33% of controls (95% CI = 29, 38) (*P* < 0.01). The percentage positive for respiratory viruses among cases was 65% from August 2013 to July 2014 with the original SARI case definition and 74% from August 2014 to July 2015 with the revised WHO SARI case definition. During the same period, the percentage positive for respiratory viruses among controls was 27% (2013–14) and 41% (2014–15) (*P* < 0.01). Detections of RSV, HMPV, influenza A virus, and PIVs (type 1 and 3) were significantly more common among cases than controls (*P* < 0.05). Among these, RSV was the most detected virus (31%; 95% CI = 28, 34), followed by hMPV (6%; 95% CI = 5, 8), PIV type 3 (5%; 95% CI = 4, 7), and influenza A virus (3%; 95% CI = 2, 4) (**Figure 1**). Among SARI cases <3 months of age, RSV was the most detected virus (n = 87, 46%; 95% CI = 39, 54%) (Figure S1 in the **Online Supplementary Document**). Detections of human coronaviruses NL63 (3%; 95% CI = 2, 3) and 229E (2%; 95% CI = 1, 3) were significantly more common among controls than cases (**Figure 1**). Among controls, human rhinoviruses had the highest detection (18%; 95% CI = 15, 22), followed by adenovirus (5%; 95% CI = 3, 7), enterovirus (4%; 95% CI = 2, 6), and RSV (3%; 95% CI = 2, 5). The association for virus detection among cases compared to controls was the strongest for RSV (aOR = 23; 95% CI = 12, 47) and lowest for influenza (type A and B combined) (aOR = 4; 95% CI = 1, 19) (**Table 3**). The EF was highest for RSV (96; 95% CI = 91, 98) and PIVs (types 1–4 combined) (86; 95% CI = 62, 95). Among SARI cases, 101 (12%) had evidence of viral co-detections, but for influenza cases, this proportion was higher (39%) (Table S2 in the **Online Supplementary Document**).

**Table 3:** Attributable aetiologic fraction for different viruses among children <5 y hospitalised with SARI at a tertiary care centre in Delhi, India (2013–15)

	Total detections, n (%)		Prevalence, % (95% CI)			
**Pathogen**	**SARI cases (n = 840)**	**Controls (n = 419)**	**SARI cases**	**Controls**	aOR (95% CI)*	**aEF (95% CI)†**
RSV	257 (30.6)	12 (2.9)	30.6 (27.5, 33.8)	2.9 (1.5, 5)	23.3 (11.6, 46.8)	96 (91, 98)
PIV	72 (8.6)	5 (1.2)	8.6 (6.8, 10.7)	1.2 (0.4, 2.8)	7.4 (2.6, 20.8)	86 (62, 95)
hMPV	51 (6.1)	5 (1.2)	6.1 (4.6, 7.9)	1.2 (0.4, 2.8)	6.1 (1.8, 20.1)	84 (44, 95)
IV	28 (3.3)	4 (1.0)	3.3 (2.2, 4.8)	1.0 (0.3, 2.4)	4.4 (1.0, 19.4)	77 (0, 95)‡
HCV	36 (4.3)	29 (6.9)	4.3 (3.0, 5.9)	6.9 (4.7, 9.8)	0.5 (0.3, 1.0)	−100 (−233, 0)

aEF – adjusted aetiologic fraction, ALRI – acute lower respiratory infection, aOR – adjusted odds ratio, CI – confidence interval, HCV – human coronaviruses, hMPV – human meta-pneumovirus, IV – influenza viruses, PIV – para-influenza viruses, RSV – respiratory syncytial virus, SARI – severe acute respiratory illness

*Adjusted for age group, gender, month of collection and viral co-detection.

†Adjusted aetiologic fraction calculated using formula aER = (aOR − 1) × 100/aOR.

‡Values lower than zero have been cut to zero.

**Figure 2:**
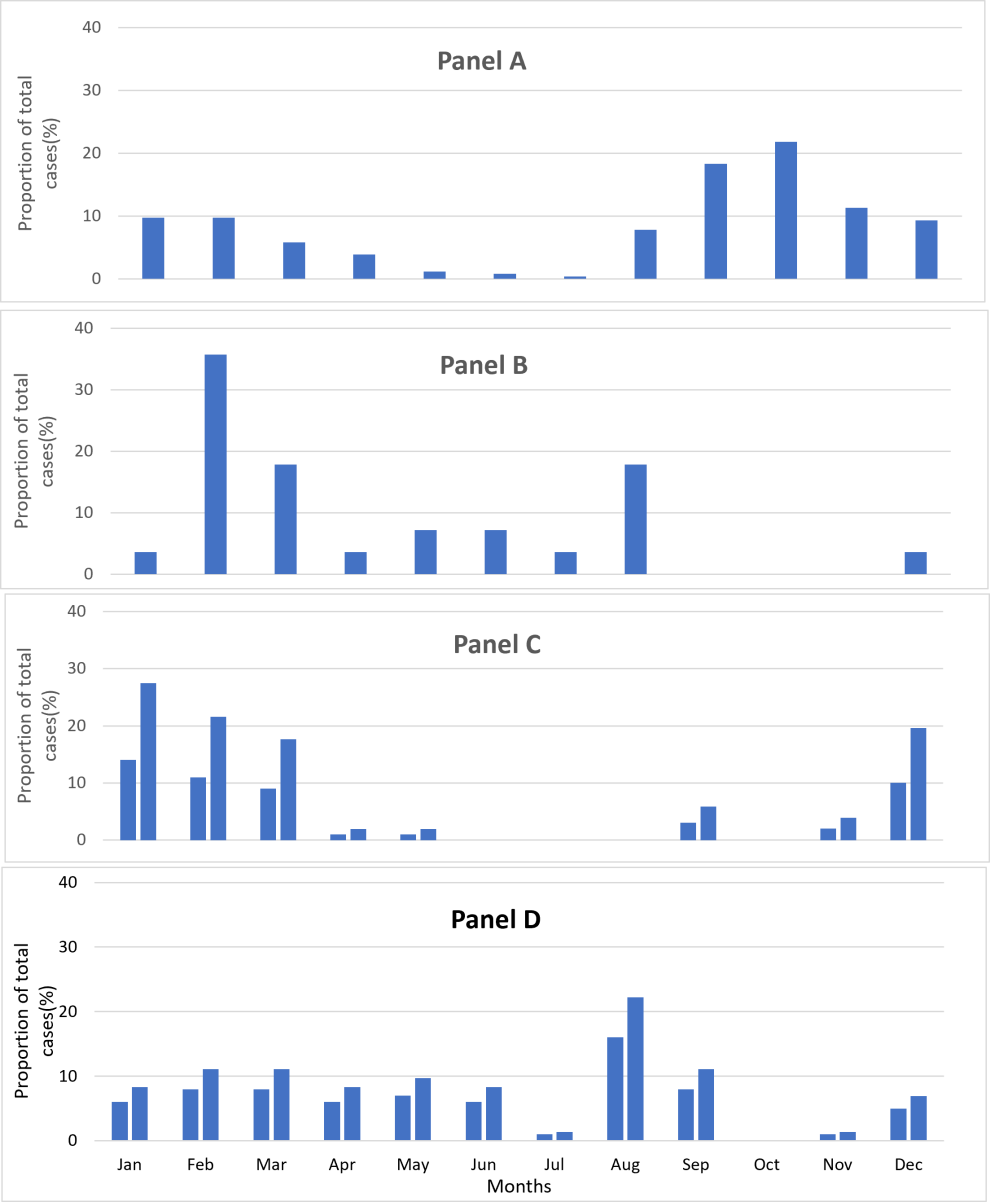
Month-wise distribution of select viruses (RSV, Influenza, hMPV and PIV) as a proportion of total cases of the respective virus detected among SARI cases aged <5 years in Delhi (2013–15). **Panel A.** RSV. **Panel B.** Influenza. **Panel C.** hMPV. **Panel D.** PIV.

During the study period (2013–15), over 80% of RSV detections were from August to February (**Figure 2**). PIVs were present throughout the year, whereas hMPV demonstrated a peak from December to March (about 85% of detections). About 90% of influenza detections occurred from February to March (54%) along with a second peak from May to August (36%).

### Treatment modalities and outcomes

Antibiotics (Table S3 in the **Online Supplementary Document**) were provided to more than three-fourths of cases (n = 651, 78%) and very few (n = 7, 1%) were treated with antivirals (i.e. oseltamivir). About half of the cases that were provided with antibiotics (n = 328, 50.4%) were given two or more antibiotics during their hospital stay. Also, more than three-fourths of cases with virus-associated SARI (n = 464, 79.6%) were given antibiotics. Bronchodilators were administered to most children (n = 758, 90%), about half of the cases (n = 393, 50%) were prescribed antipyretics and several cases were administered steroids (n = 110, 13%). More than half of cases (n = 516, 62%) were provided with supplemental oxygen and some cases were managed with mechanical ventilation (n = 19, 3%). Continuous positive airway pressure was used in three cases (0.4%) and the in-hospital case fatality was 1%, with deaths occurring in acute bronchiolitis (n = 2), pneumonia (n = 6), and cyanotic heart disease (n = 1).

## DISCUSSION

More than two-thirds of the illnesses among children aged <5 years admitted with SARI in the study hospital were associated with respiratory virus detection, adding to the evidence that respiratory viruses, especially RSV, are a common cause of hospitalisation among this population. Our study is one of a few from India that tested for common respiratory viruses among asymptomatic controls using multi-pathogen testing and estimated that the EF for RSV was the highest. Antibiotics were more commonly provided to SARI cases than antivirals despite the frequent detection of viruses.

### Comparison with earlier studies

Our findings suggest that most SARI cases among children in Delhi were associated with respiratory viruses, which aligns with previously published data from India. Two earlier studies among children <5 years with SARI in northern India have reported the proportion of cases positive for any virus as 40% (tested nine viruses) [16] and 62% (tested 15 viruses) [23]. A meta-analysis in children <5 years hospitalised for ALRI estimated the proportion of cases with virus detection at 50.4% [5]. In our study, 69% of children <5 years admitted with SARI had a detected virus, which is similar to earlier estimates of 62% [23], but higher than those reported by other studies (40 and 50.4%) [5,16]. This difference is likely due to our testing for a larger number of viruses compared to the earlier studies.

Earlier multi-country studies among children with SARI have found that RSV is a major contributor to hospitalisation [6,8,25,33]. It was also reported as the most common cause of hospitalised ALRI in one meta-analysis for children <5 years [34]. The proportion of hospitalised ALRI cases positive for RSV was 30.8% for the Southeast Asia region [35], similar to our estimate of 30.6% among SARI cases. For influenza viruses, one meta-analysis [5] estimated the proportion of children <5 years with hospitalised ALRI as 3% (95% CI = 2.2, 4.0), while another [36] calculated the proportion of influenza as 7.4% (95% CI = 6.2, 8.8) among respiratory hospitalisations of children <5 years. We estimated a similar proportion of SARI cases attributed to influenza at 3.3% (95% CI = 2.2, 4.8). In an earlier study conducted from 2009 to 2011 among children aged <5 years hospitalised with acute illness in Ballabgarh, PIVs were detected in 4%, and hMPV in 1% of children [16]. In another study from Srinagar, the proportion of PIVs and hMPV were 8% and 2%, respectively, among children aged <5 years hospitalised for SARI [23]. In our study, virus detection was 8.7% for PIVs and 6.1% for hMPV.

In terms of age distribution, most RSV detections occurred among infants, while influenza detections were more common among children aged 2–5 years. This pattern is consistent with earlier studies from India [22,23,37,38]. The range of estimates across studies could likely be due to differences in the proportion of children from different age groups, testing strategy, locations, study period, and virus seasonality. Influenza positivity varies by season, which means that the rates and proportions could differ across years of study [6,39]. Also, recent studies have indicated that while reverse transcription polymerase chain reaction is the standard method for diagnosing and detecting influenza, it may result in under-detection, especially among infants [40]. Two-thirds of our study children were infants, which likely influenced the relative positivity of the detected viruses.

An earlier global meta-analysis of 23 studies from several countries that estimated the EF of common respiratory viruses in children <5 years hospitalised with ALRI found the EF for RSV to be 90% and the EF for influenza to be 80% [34]. In comparison, we found EFs of 96% for RSV and 77% for influenza. The aforementioned meta-analysis [34] found the EFs for hMPV and PIVs to be 73% and 70%, compared to EFs of 84% for hMPV and 86% for PIV in our study. An earlier community-based study from a rural area near Delhi among children <10 years for ALRI [32] provided EFs similar to those in our study. A notable difference between our and prior studies is that, while the meta-analysis reported no significant difference in the detection of HCV in cases and controls [34], we observed such differences, with HCV detections being more common among controls.

We need to be aware of the challenges in making causal attributions here, as pointed out by a recent study in which some seemingly healthy infants who tested positive for the influenza virus became ill within a week of being swabbed [40]. The findings reported by multi-country studies such as PERCH [6] and GABRIEL [25] are largely consistent with ours, indicating that RSV was the most prevalent virus with the highest EF, especially among younger children. In our study, we enrolled outpatient controls without respiratory symptoms, similar to the approach used in GABRIEL [25], because they were readily available within the hospital environment. This approach differed from that of PERCH [6], where the investigators enrolled community controls regardless of their respiratory symptoms.

### Management of SARI

Given the high prevalence and EF of certain viruses, the fact that more than three-fourths of cases (n = 651, 77.5%) in our study were prescribed antibiotics raises concerns. Many children with virus detections had received antibiotics during their hospitalisation. Such high rates of antibiotic prescription to children with acute respiratory infections have been reported across several LMICs [8,41]. Although we could not determine the appropriateness of antibiotic prescription for each case, it is likely that antibiotics were not indicated for many of these cases, especially those that tested positive for RSV, influenza, or other viruses. The implications of high antibiotic use towards antimicrobial resistance require a more careful review of the prescription practices, including the judicious use of antivirals when appropriate.

The policy towards oseltamivir use in India changed in 2017 [42]. The guidelines from the Indian Academy of Pediatrics advise symptomatic and supportive treatment for viral pneumonia and state that ‘Oseltamivir can be given if H1N1 infection is suspected but that [treatment with Oseltamivir] should be initiated within 3 days of symptoms’ [43]. Ribavirin was licensed in India in 2015, but was seemingly too expensive, as anecdotal evidence suggests very limited usage for RSV infection [44]. Monoclonal antibodies, meanwhile, were not available until August of 2023 [45]. Knowledge of the aetiology of SARI can help facilitate appropriate management and implementation of the most effective control measures [46,47]. Co-detections and their impact on severity are poorly understood, but with increasing use of multi-pathogen tests, the data might help in better appreciation of its clinical and epidemiological significance [48,49].

Vaccines against RSV and influenza are now available for administration to pregnant mothers, providing protection for infants up to six months of age [33,50]. High-risk infants can also be provided prophylaxis against RSV with monoclonal antibodies [50,51]. For influenza, those aged ≥2 weeks can be treated with antivirals [52], with vaccines being available for children aged ≥6 months [53,54]. Additionally, ongoing research using mRNA and other techniques has been making steady progress in developing new vaccines and products, several of which have shown significant immunogenicity against other viruses such as hMPV and PIVs during early clinical trials [12,13]. Vaccines (e.g. those for influenza, rotavirus, and RSV) that help prevent febrile illness can also contribute to a reduction in antibiotic prescription and help combat antimicrobial resistance [55]. Guidelines for the prevention and management of SARI cases in children could incorporate currently available treatments, including antivirals and vaccines, to improve outcomes.

### Seasonal variations

During our study, over 80% of RSV detections occurred from August to February, which corresponds with a decrease in temperature, as reported in other research in the region [23,32,38]. Months with high influenza positivity varied over the two study years, which is consistent with the typical seasonality for tropical climate countries and has been observed in other studies describing different regions in India [32,56]. The increased data availability for viruses (e.g. prevalence and seasonality) from the Indian Council of Medical Research and Virus Research and Diagnostic Laboratories nationwide SARI surveillance networks [57] can help clinicians understand virus circulation in the population, informing appropriate prevention and treatment in clinical settings. Getting quick test results may not always be feasible in all settings; therefore, surveillance data can help clinicians prescribe antivirals and vaccines more confidently.

### Limitations

The study findings should be considered alongside their limitations. Upper respiratory tract specimens were used for testing because collecting more invasive lower respiratory specimens, which are ideal for studying aetiology, was challenging. As this can hinder causal interpretations, testing was also conducted among controls to enhance understanding of the attribution to specific pathogens [24,25,58]. About 33% of asymptomatic controls tested positive for respiratory viruses and were assumed to be representative of the general population in terms of respiratory commensals, although case-control matching was conducted at a 2:1 ratio for operational reasons. We conducted this study in a single hospital, which may have affected our ability to detect all circulating viruses in this region, therefore the findings may not be generalisable to other regions or health care settings within India or other LMICs. However, this being a prominent tertiary care centre in Delhi and adjacent areas, cases admitted in this hospital are likely to be representative of this geography. We also conducted this study before the COVID-19 pandemic, so it might not reflect the current viruses in circulation, meaning that, while viruses may continue to contribute a large proportion of SARI, the relative proportions of individual viruses could likely differ.

## CONCLUSIONS

Our findings confirm prior data on the overall high contribution of viruses towards SARI and highlight RSV as the main contributor among SARI cases in children aged <5 years in our sample. This, in turn, adds to the evidence base for enhanced use of appropriate vaccines and treatments against RSV and influenza. With newer vaccines and biologicals that are in development for common childhood respiratory viruses, optimisation of SARI management guidelines with the appropriate role of antivirals, monoclonal antibodies, and vaccines could be instrumental in lowering the burden of SARI, especially in LMICs.

## Additional material


Online Supplementary Document.
