# Supplementary material for: Respiratory viruses associated with severe acute respiratory infection in children aged <5 years at a tertiary care hospital in Delhi, India during 2013–15
Source: J Glob Health. 2024 Nov 8;14:04230. doi: 10.7189/jogh.14.04230 (PMC11544518; doi:10.7189/jogh.14.04230)
Supplement: Online Supplementary Document. [file jogh-14-04230-s001.pdf]

Supplement

Figure S1: Age-distribution of select viruses [RSV, Influenza, hMPV & PIV] detected among SARI cases aged <5 years in Delhi (2013-15) [* y axis scale is different for RSV]

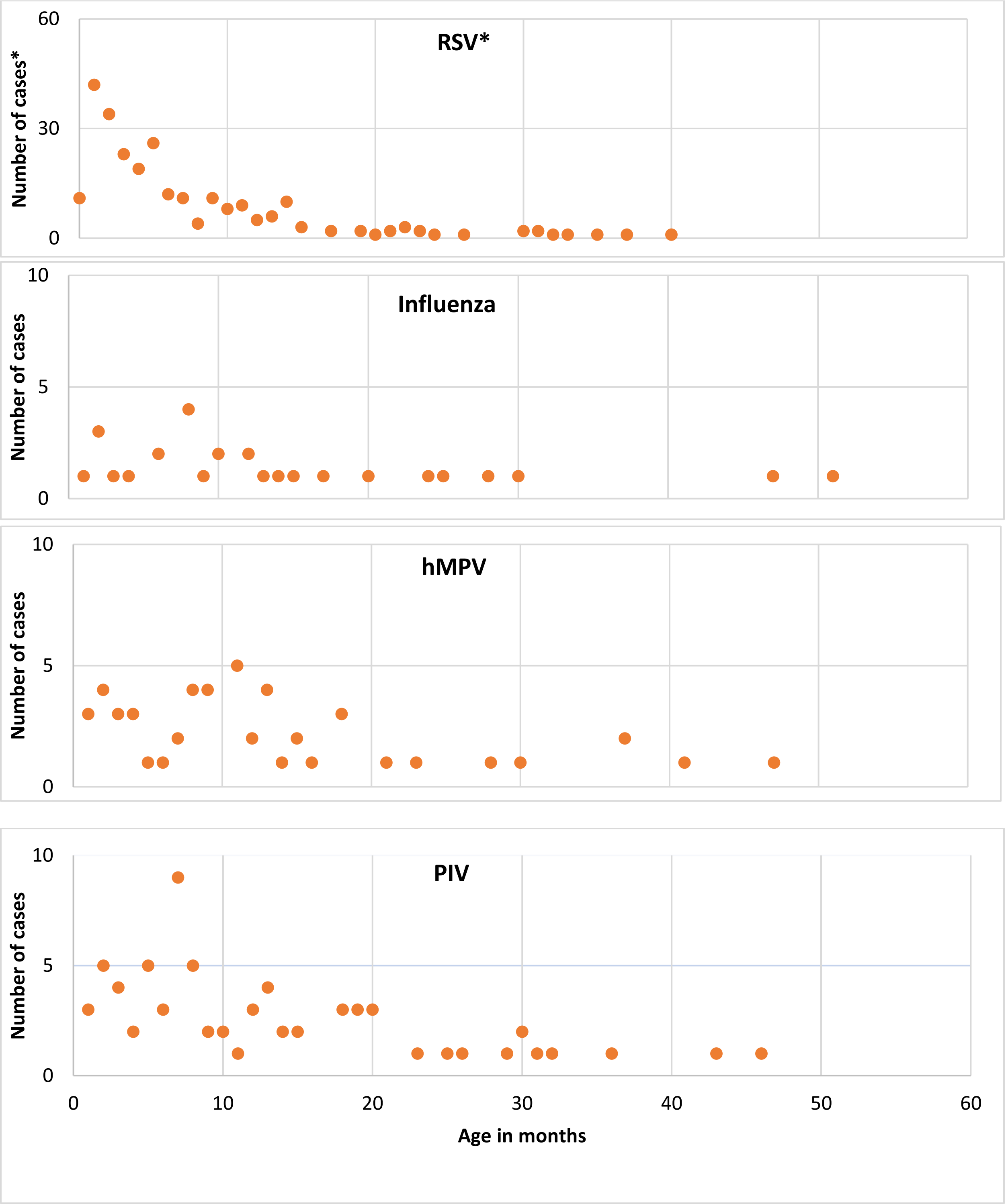

Table S1. Clinical characteristics of children <5 years hospitalized with severe acute respiratory illness (SARI) at a tertiary care center in Delhi, India (2013-15)

	PIV positive (n=72)			hMPV positive (n=51)			HCV positive (n=36)	
	n	%		N	%		N	%
**Symptoms & signs**								
Cough	72	100		51	100		36	100
Fever	69	95.8		50	98		32	88.9
Breathlessness	68	94.4		49	96.1		35	97.2
Noisy breathing	51	71.8		44	88		30	83.3
Nasal discharge/congestion	41	56.9		37	72.6		18	50
Diarrhoea	12	16.7		6	11.8		5	13.9
Earache	2	2.8		1	2		0	0
Rash	2	2.8		0	0		2	5.6
Tachypnoea	52	72.2		37	72.6		22	61.1
Wheeze	2	2.8		0	0		2	5.6
Any sign of respiratory distress[$]	58	80.6		32	62.8		30	83.3
Lethargy	61	84.7		40	78.4		24	66.7
**Clinical management**								
Antibiotics	58	80.6		37	72.6		28	77.8
Antivirals	2	2.8		0	0		0	0
Supplemental O2	48	66.7		39	76.5		23	63.9
Mechanical ventilation	1	1.4		1	2		1	2.8
Deaths (n=700)	1	1.4		0	0		1	2.8

Table S2: Co-detection of viruses among SARI cases

	RSV	Rhinovirus	hMPV	Adenovirus	Enterovirus	Influenza	PIV
**Rhinovirus**	22						
**HMPV**	5	9					
**Adenovirus**	7	14	2				
**Enterovirus**	3	15	1	1			
**Influenza**	2	2	2	1	1		
**PIV**	4	8	2	2	2	4	
**HCV**	4	3	1	1	0	2	2

Table S3: Antibiotic use among SARI cases

Name of antibiotic	No. of SARI cases prescribed/given
Amoxicillin	392
Aminoglycoside (Gentamicin)	278
Amoxicillin/Clavulanate (Augmentin)	199
Ceftriaxone	172
Vancomycin	49
Meropenem	22
Macrolide (Azithromycin, Erythromycin)	21
Piperacillin and tazobactam	14
Linezolid	3
Metronidazole	3
Colistin	2
Cotrimoxazole	2
Others*	7

*One each [Artesunate; Ciprofloxacin; Cefixime; Ceftazidime; Amphotericin B; Netilmicin; Anti TB drugs (Rifampicin, Isoniazid, Pyrazinamide, Ethambutol)]

**Antiviral (Oseltamivir) was provided to seven SARI cases (7/838=1%)
